# Memory bias in observer-performance literature

**DOI:** 10.1117/1.JMI.5.3.031412

**Published:** 2018-09-24

**Authors:** Tamara Miner Haygood, Samantha Smith, Jia Sun

**Affiliations:** a University of Texas MD Anderson Cancer Center, Department of Diagnostic Radiology, Houston, Texas, United States; b University of Texas MD Anderson Cancer Center, Department of Biostatistics, Houston, Texas, United States

**Keywords:** observer performance, observer study, memory bias

## Abstract

The objective of our study was to determine how authors of published observer–performance experiments dealt with memory bias in study design. We searched *American Journal of Roentgenology* online and *Radiology* using “observer study” and “observer performance.” We included articles from 1970 or later that reported an observer performance experiment using human observers. We recorded the methods used by the authors to order presentation of the conditions being tested and images within sets for viewing. We recorded use and length of any time gap between viewings. We included 110 experiments. Forty-five used methods not subject to memory bias. Of 68 remaining experiments, 30 (44.1%) ordered the viewing of tested conditions to decrease memory bias. Fifteen (22.1%) ordered the tested conditions in ways that may create memory bias. Eleven (16.2%) intermixed the tested conditions. Forty-three (63.2%) used random or pseudorandom ordering of images within sets. Forty-six (67.6%) used a time gap (median 14 days) between viewings. Six (8.8%) did not use a time gap. Thirty-six (52.9%) did not indicate what methods they used in at least one studied parameter. Therefore, we conclude that 22.1% of the experiments could improve their methods of ordering tested conditions. Completeness of reporting could be improved by including more details regarding methods of ameliorating memory bias.

## 1 Introduction

Observer performance studies are commonly used in radiology to determine the relative fitness of imaging techniques for a particular purpose. Typically, human observers view images that differ from one another in some way to gain insight into which might be better for a particular task. Two examples, taken from the articles considered for this paper, of research questions that may be addressed using an observer-performance study follow: (1) Does chest radiography with dual-energy subtraction images improve radiologists’ ability to find small lung cancers as compared with chest radiography without dual-energy subtraction images?^1^ (2) Does computer-aided detection improve radiologists’ interpretations of computed tomographic (CT) colonography?^2^ At other times, it may not be the images that change, but rather there is a change in their presentation or in the reading environment. Depending on the design of the experiment, observers may be asked to complete a specified task under two or more different conditions or to choose which of two or more images are best suited for a task. We have called the different image types or reading conditions that are being compared “tested conditions.”

Different experimental designs are associated with greater or lesser risk of memory bias. In alternative forced-choice designs, the observers are asked to choose the best suited among two or more tested conditions that are presented at the same time. For example, are flat-panel display monitors or high-resolution gray-scale display monitors better suited for display of anatomic features in chest radiographs? Balassy et al.^3^ addressed this question by asking observers to look at the same images projected side by side on different monitors and rank the visibility of anatomic features. In multipoint rank-order designs, an observer is asked to rank several tested conditions in order from best to worst. Sivaramakrishna et al.^4^ asked observers to compare unenhanced mammograms and the same images altered by four different enhancement algorithms, then rank them from best to worst for display of microcalcifications and masses, two findings that can indicate the presence of breast cancer. Such studies do not generally harbor a risk of memory bias. Indeed, the investigators would want the observers to remember image A when looking at image B as it is otherwise not possible to form an intelligent opinion of their relative merits.

In what we term sequential-viewing experiments, an image is viewed alone, and then immediately afterward it is seen along with an additional image or piece of information. Two examples from our reviewed articles were the study by van Rijn et al.^5^ in which observers interpreted spine MRI images alone and then with clinical information and the study by Li et al.^6^ in which observers rated the likelihood of malignancy of pulmonary nodules on CT first without and then with the results of evaluation by computer-assisted diagnosis. In sequential-viewing studies, memory bias is also not a concern as the images or other information displayed as part of the first tested condition are deliberately made available during the second interpretation. This is usually done to mimic the way the two tested conditions might be used together in actual image interpretation.

In study designs in which the tested conditions are displayed separately, memory for a previously encountered image does have the potential to create bias. In such designs, rather than looking at two CT images of the liver at the same time and choosing which one shows a tumor better, the task might be to look at them separately and try to find the liver tumor. The observers’ actual level of success at identifying liver tumors, rather than the observers’ opinion, would then indicate which type of CT image was more suitable for that task. In this type of study, if the observer remembers the first image while looking at the second image, that memory could affect the second reading and therefore bias the study.

Metz^7^ recognized the potential for bias due to recognition memory. He suggested that investigators arrange the order of readings so that any bias created by memory would cancel out. The simplest way to do this would be, in the case of an experiment comparing two different tested conditions, to have half the observers view images in tested condition A first and the other half of the observers view images in tested condition B first. This is termed counterbalanced methodology. Metz also suggested that, in addition to using such a counterbalanced approach, a time gap as long as possible should be used between viewings of the same image. Almost 20 years later, Sica^8^ made similar recommendations.

Since then, several studies have looked at the effect of time gaps between readings on memory for previously encountered images. They have found that very short time gaps of a few minutes to a few days are associated with slightly better-than-chance memory for images when observers were shown sets of single images in moderate numbers of about 20 to 40.^9^^–^^11^ A short time gap of 2 days also created memory bias for fused positron emission tomography and CT (PET/CT).^12^ A time gap of 50 days was sufficient to eliminate any conscious memory of previously viewed images.^11^ Others, using nonmedical images, have found a sharp drop in recognition memory for images over the first month after they were originally viewed, though any images that had not been forgotten in a month might still be remembered a year later.^13^^,^^14^

There has not been similar research regarding the contribution of other methods such as counterbalanced order of tested conditions as mentioned above to elimination of memory bias in observer-performance studies. In addition, it is not known what researchers are actually doing to cope with memory bias in observer-performance studies. Without this knowledge, it is difficult to consider whether any improvements in methodology may be warranted. We have studied 100 articles (110 experiments) from the online version of the journals *American Journal of Roentgenology (AJR)* and *Radiology* to determine what steps authors of published articles used to deal with potential memory bias.

## 2 Materials and Methods

### 2.1 Data Collection

We performed online searches of the journals *AJR* online and *Radiology* for the words “observer study” and “observer performance.” These searches yielded lists of articles sorted by the websites in order of relevance as determined by the internal workings of the websites themselves. Articles were eligible for inclusion if they reported an observer-performance experiment using human observers and were published in 1970 or later.

We selected articles that met inclusion criteria in order as they were presented by the websites until we had 50 articles from each journal, making a total of 100 articles. We recorded the first author’s name, the volume number, and the year in which each article was published. We then determined what steps the authors had taken to avoid recognition bias. In particular, we noted: the method used to order presentation of tested conditions to observers, method used to order the images within sets, and use of a time lapse between viewings.

We first divided methods used to order presentation of tested conditions into separate viewing, intermixed viewing, sequential viewing, one mode viewed, alternative forced choice, and multipoint rank order. Table 1 summarizes the differences between these methods. Of these six methods, only experiments using separate viewing and intermixed viewing have the potential to be affected by memory bias. Therefore, experiments that utilized other methods of presentation of tested conditions were excluded from further analysis.

**Table 1:** Study design as related to memory bias. We describe the methods as though an intrinsic feature of the images is changing between one tested condition and the next, but it could be something else such as the conditions under which viewing of the same images takes place.

Design category	Characteristics of the method	Relation to memory bias	Number of experiments using this method
Separate viewing	Tested conditions are viewed separately from one another. Typically, condition A may be viewed at one time and condition B at another. Alternatively, particularly when the differences between the tested conditions relate to the appearance of the images themselves, as opposed to something related to the reading environment, the images may be mixed together, with each image viewed separately but in a mixed order such as AABBABABAABBB…	Results for the subsequent viewings may be influenced by memory from previous viewings of the same or a similar image.	66 (60.0%)
Sequential viewing	Images are viewed first alone and then immediately afterward together with a complementary piece of information. Observers compare the image alone with the image paired with the additional information. Among the articles we reviewed, this methodology was often used to compare mammography alone versus mammography with computer-assisted detection.	Memory for the original image is not a source of bias as the original image is shown together with the complementary information on the second viewing.	22 (20%)
One mode viewed	Only one type of image is shown. The fitness of the images for a task is judged not against other images, but against some different standard such as surgical findings.	No concern for memory bias as the image is seen only once.	10 (9.1%)
Alternative forced choice	Images to be compared are shown at the same time. Observers choose which one works best for the particular task. Typically two images are compared, but the comparison can be among more than two images.	Images are directly compared, so memory for the images is a necessary component of the study.	7 (6.4%)
Multipoint rank order	Three or more images are shown at the same time. Observers rank them in order from best to worst for the particular task.	Images are directly compared, so memory for the images is a necessary component of the study.	2 (1.8%)
Not indicated	Some articles did not say whether tested conditions were viewed separately from one another.		3 (2.7%)
Total			110

We next took a more detailed look at methods used to order presentation of tested conditions for the remaining experiments. Table 2 summarizes characteristics of six methods of ordering the viewing of tested conditions.

**Table 2:** Design methods used by studies that may be subject to memory bias. This table discusses methodology for those 68 experiments that utilized a separate viewing of images or did not specify the primary organizational method.

Method of ordering tested conditions for viewing	Characteristics of the method	Relation to memory bias	Number of experiments using this method
Counter-balanced	Images of one type are viewed separately from images of another type. The order in which readers see the images alternates. Typically in an experiment with two image types, half the observers will view image type A first and the other half of the observers will view image type B first.	Limits memory bias as any advantage that one tested condition may receive by being viewed second and therefore having the benefit of any learning that occurred during the first viewing is canceled out because the other tested condition has the same advantage in a similar number of readings.	22 (32.4%)
Same order for each reader	Image type A is always viewed first followed by image type B.	This method of organization can introduce bias by treating the two image types differently. With respect to memory, the concern is that if the observer remembers the image from the first interpretation, the second viewing may have an advantage if the observer remembers the previous viewing.	14 (20.6%)
Intermixed	All tested conditions are shown in the same session, mixed together, but each image is seen separately from others.	Whether this method mitigates or promotes recognition will depend on how the individual images are arranged.	11 (16.2%)
Random	The order in which the tested conditions are shown is determined randomly. (Note that the individual images may also be sorted into sets randomly, but that is not necessary the case, even when the order in which the tested conditions are shown is determined randomly.)	Can limit memory bias. When a small number of image types and observers are involved, however, random ordering can result in an unbalanced arrangement with one image type being shown first a disproportionate number of times.	6 (8.8%)
Pseudorandom	The order in which the tested conditions are shown is determined by a method that should result in mixing of order but that is not truly random.	Can limit memory bias, and with small numbers of tested conditions and observers, pseudorandom ordering may result in a more balanced presentation of image types than actual random order.	2 (2.9%)
One set always viewed first	Unique ordering method fitting only one experiment. The experiment studied the value of axial MRI images alone versus axial images plus one of the two types of coronal MRI images. All observers viewed the axial images first, then they viewed the axial images with a coronal image. The order in which the two different coronal images were paired with the axial images was intermixed. All coronal images of both types were shown in the same viewing session.	In this case, intermixed viewing of the coronal images should cancel out any memory-related advantage of one type of coronal image over the other, but the combination of axial and coronal images could have an advantage over the axial images alone due to consistently being shown second.	1 (1.5%)
Not indicated	Some articles did not say how viewing of the tested conditions was ordered beyond indicating that they were viewed separately.		12 (17.6%)
Total			68

We recorded the method used to order individual images within sets for viewing. The methods used were random or pseudorandom ordering. By pseudorandom, we mean ordering that will effectively mix up the cases but that is not truly random. An example from our articles is a study of magnetic resonance imaging for detection of tears of the knee menisci. Images were taken from patients and were shown in alphabetical order according to the patient’s last name. Alphabetical order is not random, but it should effectively mix together the images with and without meniscal tears as there is no association between the first few letters of a patient’s name and the likelihood that the patient will have a meniscal tear.^15^ If the authors asserted a method to be random, the method was recorded as random, even if some constraints to random ordering were specified.^16^

We recorded whether the authors used a time gap between viewing of image types, and we recorded the length of the time lapse in days. When the lapse in time was reported as a range, we used the lower end of the range.

### 2.2 Statistical Methods

Time lapse was summarized in terms of mean, median, and range. The year of publication and other characteristics were presented as counts and percentages. Correlation between methods used in the included articles and decade of publication was tested using the exact Cochran–Armitage trend test.

## 3 Results

Data are presented with reference to the number of experiments analyzed. We reviewed 50 articles in *AJR*, in which 57 separate experiments were reported, and 50 articles in *Radiology*, in which 53 experiments were reported. Therefore, the total number of experiments was 110. Year of publication ranged from 1973 to 2016. Seven experiments (10.3%) were published between 1973 and 1979; 9 (13.2%) between 1980 and 1989; 9 (13.2%) between 1990 and 1999; 25 (36.8%) between 2000 and 2009; and 18 (26.5%) between 2010 and 2016.

The methods by which tested conditions were organized for presentation to observers are shown in Table 1. Separate viewing, in which the tested conditions are viewed apart from one another, was the most common choice and was used in 66 experiments (60.0%). Forty-one (37.3%) experiments used methods of ordering of tested conditions that are not sensitive to memory bias and therefore were not included in additional analysis. Three experiments (2.7%) did not indicate how tested conditions were ordered for viewing, and one of these experiments was excluded because comparison of very different-appearing modalities (MRI and ultrasound) made memory bias quite unlikely. This left 68 experiments for further analysis, 32 from *AJR*, and 36 from *Radiology*, respectively.

Within these 68 experiments, method of ordering of tested conditions can be evaluated in further detail as in Table 2. The most common subcategory was counterbalanced ordering, in which order of viewing of tested conditions is deliberately alternated. Twenty-two (32.4%) experiments used this method. Adding together those 22 experiments plus 6 that used random ordering and 2 that used pseudorandom ordering give a total of 30 (44.1%) experiments that used a method that will mitigate memory bias. Eleven (16.2%) experiments intermixed the tested conditions. This method may or may not mitigate memory bias depending on how the individual images were ordered for viewing within sets. Fifteen (22.1%) showed at least 1 tested condition in the same order to all viewers. Twelve (17.6%) experiments did not provide sufficient detail for further characterization.

Among these 68 experiments, ordering within a set of images to be viewed in one observation session was random in 40 (58.8%) experiments. Three (4.4%) experiments ordered them using a pseudorandom method. Reports for 25 (36.8%) experiments did not indicate how the images were ordered within sets.

Correlation between the decade in which the experiment was reported and either the methods used to order tested conditions for viewing or the methods used to order images within sets did not reveal any statistically significant difference over time.

Fifty-two of the 68 experiments (76.5%) indicated whether a time lapse was used between viewings. Six (8.8%) experiments did not use a time gap. Time gaps in experiments that utilized them ranged from 1 day for 6 (8.8%) experiments to 730 days for 1 (1.5%) experiment. Average time lapse was 36.6 days (40.5 days if those without a time lapse are excluded from the calculation). Median time lapse was 14 days whether calculated with or without the six experiments without a time lapse. The most commonly used time lapse was 7 days, used in 8 (11.8%) experiments, followed by 1 day, 14 days, and 28 days, each used in 6 (8.8%) experiments. Sixteen (23.5%) experiments did not indicate if a time lapse was used. (See Table 3)

**Table 3:** Time gap in days between viewings of the same or similar images.

Time gap in days	Number of experiments
0	6
1	6
3	3
7	8
12	1
14	6
21	3
28	6
30	4
35	1
42	1
60	3
90	2
180	1
730	1
Not indicated	16
Total	68

Reports for 24 (35.3%) of the 68 experiments did not indicate how the authors dealt with 1 of the 3 design elements we studied—either use or nonuse of a method of ordering tested conditions that would mitigate memory bias, method of ordering of images within sets, or use of a time gap. Reports for 7 (10.3%) of the 68 experiments did not indicate how the authors dealt with 2 out of the 3 design elements. Reports for 5 (7.4%) of the 68 experiment did not indicate how the authors dealt with any of the 3 design elements. Therefore, reports for 36 (52.9%) experiments did not indicate how authors dealt with at least 1 of the 3 elements.

## 4 Discussion

Metz^7^ and Sica,^8^ writing on design of imaging studies, both urged investigators to adopt methods that can mitigate memory bias. Our results showed that 22.1% of the 68 studies that were potentially affected by memory bias did not use methods of ordering the tested conditions that would decrease memory bias. The methods section of scientific papers should describe the research plan in sufficient detail that the experiment could be reproduced if desired. The STARD guidelines, both as originally published in 2003 and as updated in 2015, emphasize the need to describe methods clearly, with part of the intent being to allow readers to determine what sorts of bias may exist in the results.^17^^,^^18^ Nonetheless, we found that over half of the reports of these experiments left unstated how the authors dealt with at least one design element significant to memory bias.

We suggest that improvements may be made with respect to memory bias both in designing experiments and reporting them. Whenever possible, investigators using an experimental technique that is subject to memory bias should choose design components that will mitigate such bias including, for example, counterbalanced presentation of tested conditions, random ordering of images within sets, and use of a time gap between viewings of the same or similar images. The methods used should then be reported. A study of detection of pulmonary nodules on chest radiography by Szucx-Farkas et al.^19^ is a good example of such reporting. The methods by which the tested conditions were ordered for viewing and the images were ordered within sets, and the use of a time gap were all set forth in one paragraph.

Like all studies, ours has limitations. One is that even small changes in the design of the study could have resulted in inclusion of a different assortment of articles and therefore have altered results. For example, we wished to include articles from two old, established journals that include reports on a wide variety of radiological topics. Although we believe that *Radiology* and *AJR* online were good choices, different journals could have been chosen. Another is that the variations brought by investigators to the design of their experiments were many and imaginative. We could not discuss 110 experiments individually and therefore had to group them together into categories depending on various aspects of experimental design. It is possible that not everyone would agree in every instance with our final decision as to which category might fit a particular experiment best. Nonetheless, we believe that we have gathered a representative sample of published articles and have garnered a good sense of what authors of observer-performance studies are doing to manage subjects’ memory of images. Furthermore, any investigators attempting this research would face the same challenges and therefore have the same limitations.

## 5 Conclusions

We urge that reports of observer-performance studies that involve repeated viewing of individual images or closely related images should include details relevant to handling of observers’ memory. Specifically, they should indicate how they organized both the tested conditions and the images within sets for viewing. They should indicate if a time gap was utilized and, if so, how long it was. Including this information will allow other researchers to accurately replicate previous studies’ experimental procedures and will allow readers to judge whether memory bias was likely to have played a role in the results.

In designing observer-performance experiments, we would recommend that experimenters should minimize memory bias when observers will be viewing the same image (or closely related images) more than once (and not in a sequential-viewing methodology). This can be done by showing the images in different and random orders across viewings and observers. Entire sets of images can be alternated in a counterbalanced fashion to cancel out the memory effect that might occur if all observers saw the same tested condition before the other. Individual images can also be alternated in a more dynamic fashion so that observers encounter different assortments of images during different parts of the experiment. If experimental conditions allow for a time gap, seven weeks should be adequate between viewings, and if other memory-mitigating steps are taken, even without a gap, conscious memory for images should play a minor role.^9^^–^^11^
